# *Clonorchis sinensis* Co-infection Could Affect the Disease State and Treatment Response of HBV Patients

**DOI:** 10.1371/journal.pntd.0004806

**Published:** 2016-06-27

**Authors:** Wenfang Li, Huimin Dong, Yan Huang, Tingjin Chen, Xiangzhan Kong, Hengchang Sun, Xinbing Yu, Jin Xu

**Affiliations:** 1 Department of Parasitology, Zhongshan School of Medicine, Sun Yat-sen University, Guangzhou, Guangdong, China; 2 Key Laboratory for Tropical Disease Control, Sun Yat-sen University, Ministry of Education, Guangzhou, Guangdong, China; 3 Department of Clinical Laboratory, Third Affiliated Hospital, Sun Yat-Sen University, Guangzhou, Guangdong, China; Seoul National University College of Medicine, KOREA

## Abstract

**Background:**

*Clonorchis sinensis* (*C*. *sinensis*) is considered to be an important parasitic zoonosis because it infects approximately 35 million people, while approximately 15 million were distributed in China. Hepatitis B virus (HBV) infection is a major public health issue. Two types of pathogens have the potential to cause human liver disease and eventually hepatocellular carcinoma. Concurrent infection with HBV and *C*. *sinensis* is often observed in some areas where *C*. *sinensis* is endemic. However, whether *C*. *sinensis* could impact HBV infection or vice versa remains unknown.

**Principal Findings:**

Co-infection with *C*. *sinensis* and HBV develops predominantly in males. Co-infected *C*. *sinensis* and HBV patients presented weaker liver function and higher HBV DNA titers. Combination treatment with antiviral and anti-*C*. *sinensis* drugs in co-infected patients could contribute to a reduction in viral load and help with liver function recovery. Excretory-secretory products (ESPs) may, in some ways, increase HBV viral replication *in vitro*. A mixture of ESP and HBV positive sera could induce peripheral blood mononuclear cells (PBMCs) to produce higher level of Th2 cytokines including IL-4, IL-6 and IL-10 compared to HBV alone, it seems that due to presence of ESP, the cytokine production shift towards Th2. *C*. *sinensis*/HBV co-infected patients showed higher serum IL-6 and IL-10 levels and lower serum IFN-γ levels.

**Conclusions/Significance:**

Patients with concomitant *C*. *sinensis* and HBV infection presented weaker liver function and higher HBV DNA copies. In co-infected patients, the efficacy of anti-viral treatment was better in patients who were prescribed with entecavir and praziquantel than entecavir alone. One possible reason for the weaker response to antiviral therapies in co-infected patients was the shift in cytokine production from Th1 to Th2 that may inhibit viral clearance. *C*. *sinensis*/HBV co-infection could exacerbate the imbalance of Th1/Th2 cytokine.

## Author Summary

Clonorchiasis and hepatitis B infection are infectious diseases that affect millions of people worldwide, especially in China. These two diseases are caused by two different pathogens, *C*. *sinensis* and hepatitis B virus, respectively. Concurrent infection between HBV and *C*. *sinensis* is often observed in some areas where *C*. *sinensis* is endemic. Both diseases share the same target organ, but there is little known on whether concomitant clonorchiasis could have an impact on HBV infection and the efficacy of antiviral treatment. In this study, we showed for the first time that co-infection with *C*. *sinensis* and HBV resulted in significantly higher liver transaminases levels as well as HBV DNA copies, indicating that co-infection with *C*. *sinensis* and HBV infection may aggravate the disease state. Combination treatment with antiviral and anti-*C*. *sinensis* drugs in co-infected patients could contribute to a reduction in viral load and help with liver function recovery. Furthermore, excretory-secretory products (ESPs) of *C*. *sinensis* may have a potential role in promoting HBV viral replication. This may explain, at least in part, the higher HBV DNA copies observed in co-infected patients. Additionally, a mixture of ESP and HBV positive sera could induce PBMCs to mainly produce Th2 cytokines such as IL-4, IL-6 and IL-10 compared to HBV alone. A possible reason for higher HBV DNA copies and a weaker response to antiviral therapies in co-infected patients was the shift in cytokine production from Th1 to Th2 that may inhibit viral clearance.

## Introduction

Clonorchiasis, caused by *Clonorchis sinensis* (*C*. *sinensis*), is one of the parasitic zoonosis. It is estimated that approximately 35 million people are infected in Asia, among which approximately 15 million infected people were in China [1–3]. Previous epidemiological data showed that clonorchiasis is endemic in the southeast of China, especially in Guangdong province [4]. The people become infected with *C*. *sinensis* by the consumption of raw or undercooked fish that contains *C*. *sinensis* metacercariae [5]. The adult worms of *C*. *sinensis*, located in the small bile ducts of the liver, lead to mechanical damage, while excretory-secretory products (ESPs) of *C*. *sinensis* cause chemical damage. Both mechanical damage and chemical damage play a key role in causing hyperplasia and adenomatous changes in the bile ducts[6]. The ESPs are known to be involved in parasite-host interaction and have clinical significance in the diagnosis and pathogenesis [7–10].

Hepatitis B virus (HBV) infection is a major public health issue that may develop into cirrhosis, hepatic decompensation and hepatocellular carcinoma (HCC)[11]. An estimated 2 billion people have been infected, and more than 350 million are chronic carriers of the virus despite the availability of a prophylactic vaccine[12]. HBV infection situation is even more severe in China where approximately 170 million people are chronically infected with HBV [13,14]. Th1 responses seem to be involved in the clearance of HBV, while chronic HBV infection elicits very weak T cell responses [15,16].

A relatively smaller population is infected with *C*. *sinensis* compared with HBV[17]; concurrent infection with HBV and *C*. *sinensis* is often observed in areas where *C*. *sinensis* is endemic[18]. In some epidemiological studies, the positive rate of HBV surface-antigen (HBsAg) was significantly higher in areas endemic with *C*. *sinensis* than non-endemic areas [19–21]. However, *C*. *sinensis* infection and chronic HBV infection are two different causes of liver disease in China, and limited data are currently available as to whether there is any association between HBV and *C*. *sinensis* infections. There has been no clear discussion as to whether the *C*. *sinensis* infection may impact upon HBV infection or vice versa. The aim of our study is to evaluate the impact of *C*. *sinensis* infection on HBV infection as well as the response to antiviral therapy in co-infection with *C*. *sinensis* and HBV. Our results showed that co-infected individuals presented weaker liver function and higher HBV DNA titers. In co-infected patients, the efficacy of anti-viral treatment was better in patients who were prescribed ETV and PZQ than ETV alone. A possible reason for higher HBV DNA copies and a weaker response to antiviral therapies in co-infected patients was the shift in cytokine production from Th1 to Th2 that may inhibit viral clearance.

## Methods

### Ethical statement

The Institutional Review Boards of the Third Affiliated Hospital and Zhongshan School of Medicine, Sun Yat-sen University, approved this study as an exempt study for which informed consent did not need to be sought from subjects. Informed consent was not sought for this study as all information was obtained from the existing medical record, and data were analyzed anonymously.

HBV positive sera were obtained from chronic HBV patients, and peripheral blood mononuclear cells (PBMCs) were collected from healthy donors or chronic HBV patients from the Third Affiliated Hospital of Sun Yat-sen University. Samples were anonymously coded in accordance with local ethical guidelines (as stipulated by the Declaration of Helsinki), and written informed consent was obtained from patients and healthy volunteers. The work was conducted in strict accordance with the study design as approved by the Clinical Research Ethics Committee of the Third Affiliated Hospital and Zhongshan School of Medicine, Sun Yat-sen University, Guangzhou, China.

### Patients

First, we evaluated HBV patients by screening all consecutive patients at the Third Affiliated Hospital of Sun Yat-sen University between July 2014 and February 2015. The inclusion criteria for patients who were mono-infected with HBV were the following: men and women aged 18 years and older; HBV surface-antigen (HBsAg)-positive; and HBV DNA >20 IU/mL. The inclusion criteria for patients who were co-infected were the following: men and women aged 18 years and older; HBV surface-antigen (HBsAg)-positive; HBV DNA >20 IU/mL; and having *C*. *sinensis* eggs in the stools. The inclusion criterion for patients who were mono-infected with *C*. *sinensis* was having *C*. *sinensis* eggs in the stools. The inclusion criteria for healthy subjects were negative for both HBV and *C*. *sinensis*. Furthermore, we compared co-infected patients who were treated with entecavir (ETV, 0.5 mg once daily) alone with those who received a combination treatment of ETV (0.5 mg once daily) and praziquantel (PZQ, 210 mg/kg, 3 times a day, for 3 days) to determine the impact of *C*. *sinensis* on antiviral therapies. The following data were collected from electronic medical records by computer-assisted chart review: age, gender, date of prescription of antiviral drug ETV and PZQ drug, HBV DNA copy numbers, serial liver function test once in 2 weeks, including aspartate aminotransferase (AST), alanine aminotransferase (ALT), and total bilirubin (TB). Patients with the following concomitant conditions were excluded: those co-infected with HIV, hepatitis A, C, D and E, those with type I and type II diabetes, those co-infected with *Schistosoma japonicum*, or *Schistosoma mansoni* or other parasites, and those with alcoholic liver, autoimmune diseases, cholestasis, serious heart diseases and pregnant women. Due to the retrospective nature of the study, written informed consent could not be obtained from all patients. All data were de-identified prior to analysis.

### Biochemical and serological testing

Biochemical tests were performed using routine automated analyzers. HBsAg was detected by electrochemiluminescence immunoassay with COI >1.00 (COBAS). Serum ALT, AST, and TB levels were determined using commercial kits (Maccura, China). Serum levels of HBV DNA were measured by real-time PCR with a lower detection limit of 20 IU/mL (COBAS).

### Preparation of ESPs, PBMCs and cell cultures

ESPs were obtained as previously described [22]. Briefly, the living adult *C*. *sinensis* parasites were cultured in DMEM, and the supernatant was collected at 48 h.

Fresh whole blood (5 ml) was obtained from health adult volunteers and chronic HBV patients. The blood was mixed with the same volume of phosphate-buffered saline (PBS) and layered on 5 ml of lymphocyte separation medium (TBD, China). The sample was centrifuged at 2000 rpm for 20 min at RT. PBMCs from the upper portion of the Ficoll layer were collected, washed with PBS and centrifuged at 1500 rpm for 10 min at RT. The PBMCs were suspended in RPMI 1640 (Gibco) with 10% fetal bovine serum (Gibco) at a concentration of 2 × 10^6^ cells/ml in endotoxin-free tubes.

PBMCs, seeded into 12-well cell culture clusters at a density of 1.0 × 10^6^ viable cells per 200 μl of culture medium, were incubated with one of the following conditions: 1) HBV DNA-positive sera (2.0 ×10^6^ HBV DNA IU/mL), 2) HBV DNA-positive sera (2×10^6^ HBV DNA IU/mL) and ESPs (20 μg/mL), or 3) ESPs (20 μg/mL) alone for 48 h at 37°C in a humidified 5% CO_2_ incubator. Finally, cells from each culture and their corresponding supernatants were analyzed cytokine mRNA expression and HBV DNA copies, respectively.

### Real-time reverse transcription polymerase chain reaction (RT-PCR) analysis

Total RNA of PBMCs was extracted using Trizol reagent (Life Technologies, USA) according to the manufacturer’s protocol. cDNAs were synthesized using a cDNA Synthesis Kit (TransGen, China). Quantitative real-time PCR was performed using a Bio-Rad CFX96 Real-Time System (Bio-Rad, USA) to measure SYBR Green (TRANSGEN BIOTECH, China) incorporation into double stranded amplicons. Reactions were performed in 20 μl volumes containing forward and reverse primers at a final concentration of 100 nM. Primer sequences are listed in Table 1. The PCR reaction conditions included a denaturation step at 94°C for 30 sec, then 40 cycles of a three-step cycling reaction as follows: 94°C for 5 sec, then 55°C for 15 sec and 72°C for 10 sec. Melting curve analysis revealed a single peak for each primer set. IL-2, IL-4, IL-6, IL-10 and IFN-γ were measured and normalized relative to β-actin expression. The changes in mRNA expression were analyzed by calculating 2^-ΔΔCt^. The accession numbers for genes mentioned in the text are listed in S1 Table.

**Table 1:** Primer sequences for quantitative real-time PCR.

Gene	Primer sequence 5'-3'
Human IL-2	
Forward primer	ATGTACAGGATGCAACTCCTGTCTT
Reverse primer	GTCAGTGTTGAGATGATGCTTTGAC
Human IFN-γ	
Forward primer	TGAATGTCCAACGCAAAGCA
Reverse primer	ACTCCTTTTTCGCTTCCCTGT
Human IL-4	
Forward primer	CCTCTGTTCTTCCTGCTAGCA
Reverse primer	GCCGTTTCAGGAATCGGATCA
Human IL-6	
Forward primer	ATGAACTCCTTCTCCACAAGCGC
Reverse primer	GAAGAGCCCTCAGGCTGGACTG
Human IL-10	
Forward primer	GCACAGCTCAGCACTGCTCTGTTG
Reverse primer	TCAGTTTCGTATCTTCATTGTCATGTA
Human β-actin	
Forward primer	CACTCTTCCAGCCTTCCTTCC
Reverse primer	CGGACTCGTCATACTCCTGCTT

### Cytokine assays

Serum samples were obtained by centrifugation at 3000 rpm for 5 min. Serum samples were immediately stored at -80°C and thawed prior to analysis. Cell culture supernatants and serum concentrations of IL-2, IL-4, IL-6, IL-10 and IFN-γ were analyzed by ELISA with commercially available kits (Elabscience, China) according to the manufacturer’s instructions. The concentration of each cytokine was determined using a standard curve according to the kit instructions.

### Statistical analysis

All data were presented as the mean values±standard error or mean values. Data analyses were carried out using the GraphPad Prism software 5.0. For comparison with more than two groups, one-way ANOVA test was conducted, and if the data were nonparametric, a Kruskal-Wallis test with a confidence interval of 95% was employed. *p*<0.05 was considered statistically significant.

## Results

### Co-infected patients present weaker liver function and higher HBV DNA copy numbers

During the study period, there were 701 patients who met the selection criteria, of whom 51 were *C*. *sinensis*/HBV co-infected, 53 were *C*. *sinensis* mono-infected and 520 were HBV mono-infected. In addition, 77 healthy individuals with a mean age similar to the patient population were included. The patients' characteristics are reported in Table 2. Patients in the infected groups were predominantly men (94% for *C*. *sinensis*/HBV co-infected, 73% for mono-HBV, 81% for *C*. *sinensis* infected). Liver function was assessed by measurement of TB, ALT and AST in plasma. All 3 infected groups of patients showed higher levels of ALT, AST and TB than the healthy control subjects. ALT, AST and TB levels were significantly higher in the co-infected group (*p*<0.0001, *p*<0.0001, *p*<0.0001, respectively) compared to HBV mono-infected patients. Furthermore, HBV DNA log copies were also significantly higher in the co-infected patient group (*p* <0.05, Table 2). Taken together, these data indicate that patients co-infected with *C*. *sinensis* and HBV had weaker liver function than mono-HBV infected and that the presence of *C*. *sinensis* may aggravate the disease state.

**Table 2:** Clinical characteristics of the study cohort.

	Co-infected (*C*. *sinensis*/HBV) (n = 51)	Mono HBV infected (HBV) (n = 520)	Mono-*C*. *sinensis* infected (n = 53)	Healthy controls (n = 77)
Gender				
Male	48 (94%)	353 (68%)	43 (81%)	45 (58%)
Female	3 (6%)	167 (32%)	10 (19%)	32 (42%)
Age (years)				
Mean ± SD	45.31±1.393	36.84±0.4956	47.61±1.677	41.48±1.548
≤50 years (n)	33 (65%)	311 (60%)	34 (58%)	58 (75%)
>50 years (n)	18 (35%)	209 (40%)	25 (42%)	19 (25%)
ALT (U/L)	353.5±89.11^a^^b^^c^	89.33±7.654^a^^c^	38.49±3.979	24.14±0.9606
AST (U/L)	247.9±67.67^a^^b^^c^	66.00±5.256^a^^c^	35.13±2.770	24.52±0.9376
TB (μmol/L)	139.2±21.53^a^^b^^c^	36.96±4.458^a^	46.48±13.83	12.84±0.7046
HBV DNA copies, log (IU /mL)	5.495±0. 2229^b^	4.952±0.07978	negative	negative

Liver function was evaluated by measuring the levels of TB, AST and ALT. *C*. *sinensis* infection was diagnosed by counting *C*. *sinensis* eggs in stool specimens. Data are expressed as the mean±SD. AST: aspartate aminotransferase; ALT: alanine aminotransferase; HBV: hepatitis B virus; TB: total bilirubin

^a^ Statistically significantly different vs. healthy control subjects

^b^ Statistically significantly different vs. HBV mono-infected patients

^c^ Statistically significantly different vs. *C*. *sinensis* mono-infected patients

### Impact of anti-clonorchiasis treatment on antiviral therapies in co-infected patients

**Fig 1:**
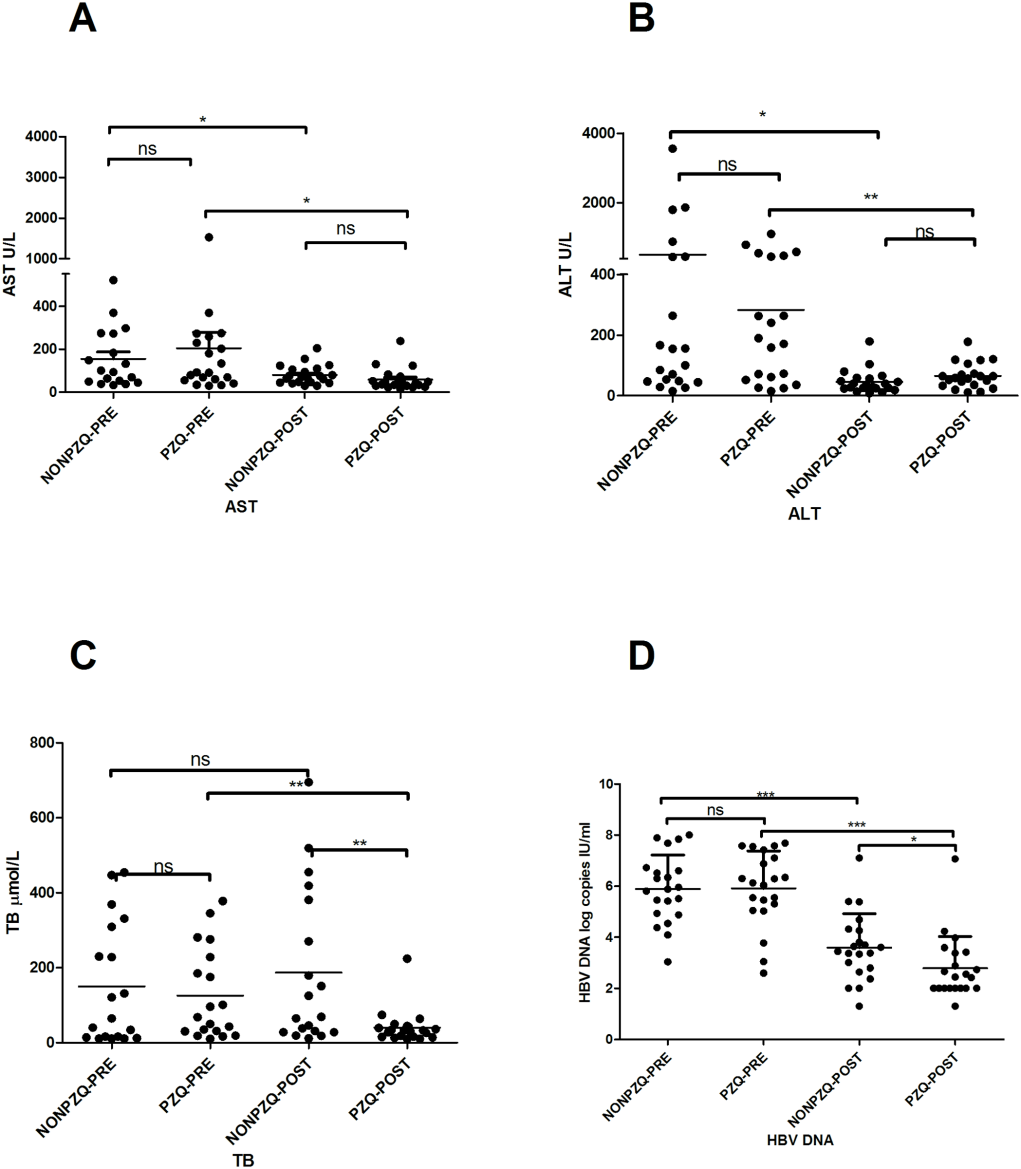
Impact of anti-*C*. *sinensis* treatment on antiviral therapies in co-infected patients. Elevated liver transaminases (including AST, ALT and TB) and HBV DNA copies in the plasma of co-infected patients were detected after receiving combination treatment ETV and PZQ or not. (A) AST and (B) ALT and (C) TB were measured in plasma. (D) HBV DNA levels were measured by RT-PCR. Symbols show individual measurements within the patient groups, and the graphs show the means ± SD. Asterisks indicate statistically significant differences between NONPZQ and PZQ groups, as measured by paired, two-tailed Student's t-test (*p <0.05, ** *p* <0.01)

Given that the presence of *C*. *sinensis* may aggravate HBV infection disease state, we further investigated whether inhibition of *C*. *sinensis* could influence the efficacy of antiviral treatment in co-infected patients clinically. There were 51 co-infected patients, 21 of whom were prescribed ETV and PZQ drugs and 30 of whom were prescribed antiviral ETV drugs only. Because 9 out of 30 patients had not been checked for liver function after treatment, they were excluded from this part of the study. There were no significant differences in HBV DNA copies and the levels of ALT, AST and TB between the two groups before treatment (Fig 1). After one program of treatment, the level of ALT, and AST and HBV DNA copies were significantly decreased compared to the pretreatment values in both groups, but no significant differences were observed in the levels of ALT and AST between the two groups (Fig 1A and 1B). There was no obvious change in TB level between pre- and post-treatment in *C*. *sinensis*/HBV-NONPZQ groups. However, *C*. *sinensis* /HBV-PZQ patients demonstrated significantly lower levels of TB than *C*. *sinensis* /HBV -NONPZQ patients after one program of treatment (*p*<0.01, Fig 1C). Additionally, *C*. *sinensis* /HBV-PZQ patients had lower levels of HBV DNA log copies compared to *C*. *sinensis* /HBV–NONPZQ (*p* <0.05, Fig 1D). Patients who took PZQ showed *C*. *sinensis* eggs negative by Kato-Katz thick stool smear technique (S1 Fig). Together, these results suggested that combined antiviral and anti-clonorchiasis drugs in co-infected patients could contribute to a reduction in viral load and help with liver function recovery.

### Influence of *C*. *sinensis* ESPs on HBV propagation in PBMCs

**Fig 2:**
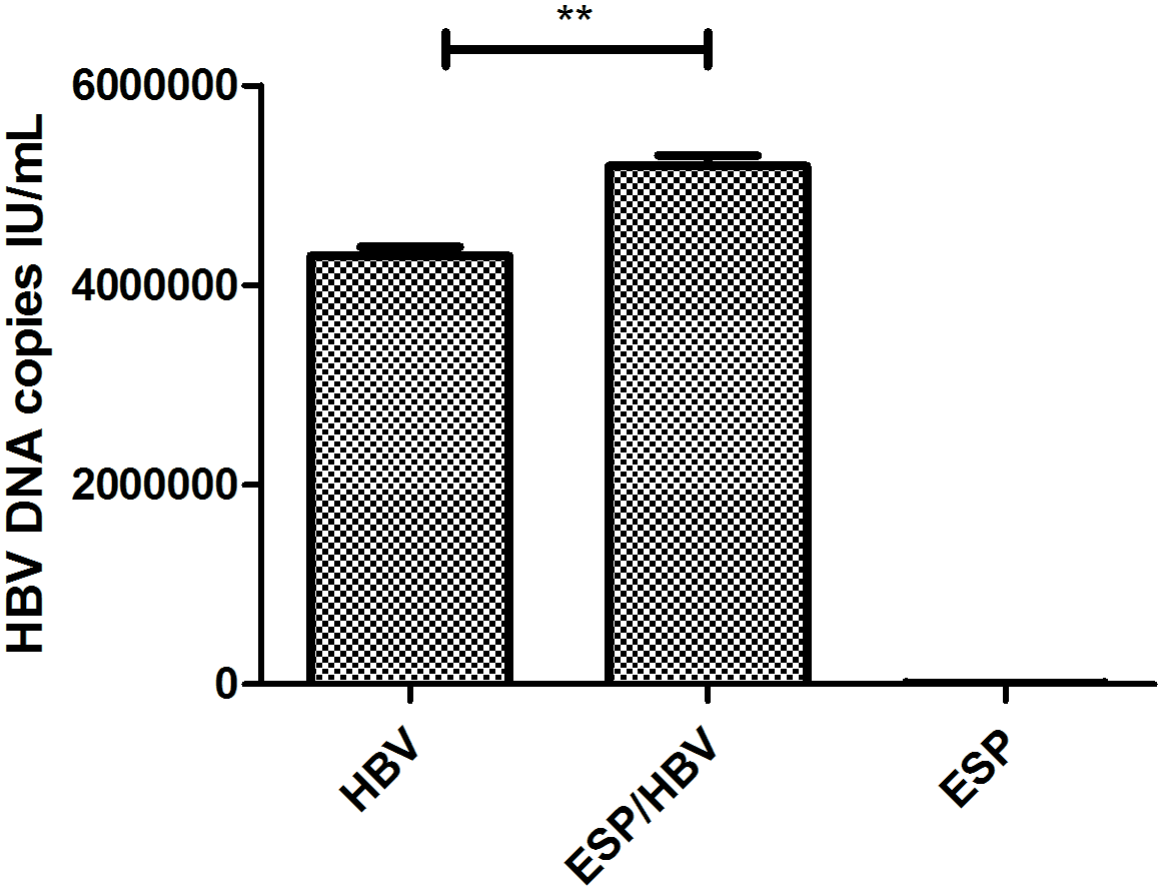
Detection of HBV-DNA copies in PBMC culture supernatant. HBV DNA was detected by FQ-PCR in the supernatant of the PBMCs incubated with mixtures of ESP and HBV positive serum or HBV positive serum only, or ESPs only. Medium alone served as a control. Asterisks indicate statistically significant differences between HBV positive sera only and mixtures of HBV positive sera and ESPs, as measured by paired, two-tailed Student's t-test (** *p* <0.01).

Co-infected patients not only showed weaker liver function but also had significantly higher HBV DNA copies clinically. We reasoned that some metabolites of *C*. *sinensis* may directly enhance HBV replication. To address this possibility, we tested HBV DNA copies in the supernatants after co-cultured of PBMCs with ESP and HBV positive patient sera. HBV positive patient serum alone and ESPs alone served as controls. HBV DNA was measured by real-time PCR with a lower detection limit of 20 IU/mL. As expected (Fig 2), HBV DNA copies were significantly higher in the culture supernatants from the PBMCs co-cultured with ESP and HBV mixture than the control groups (*p* <0.01). These data suggested that ESPs may, in some ways, promote viral replication.

### mRNA levels of cytokine secreted by stimulated PBMCs

**Fig 3:**
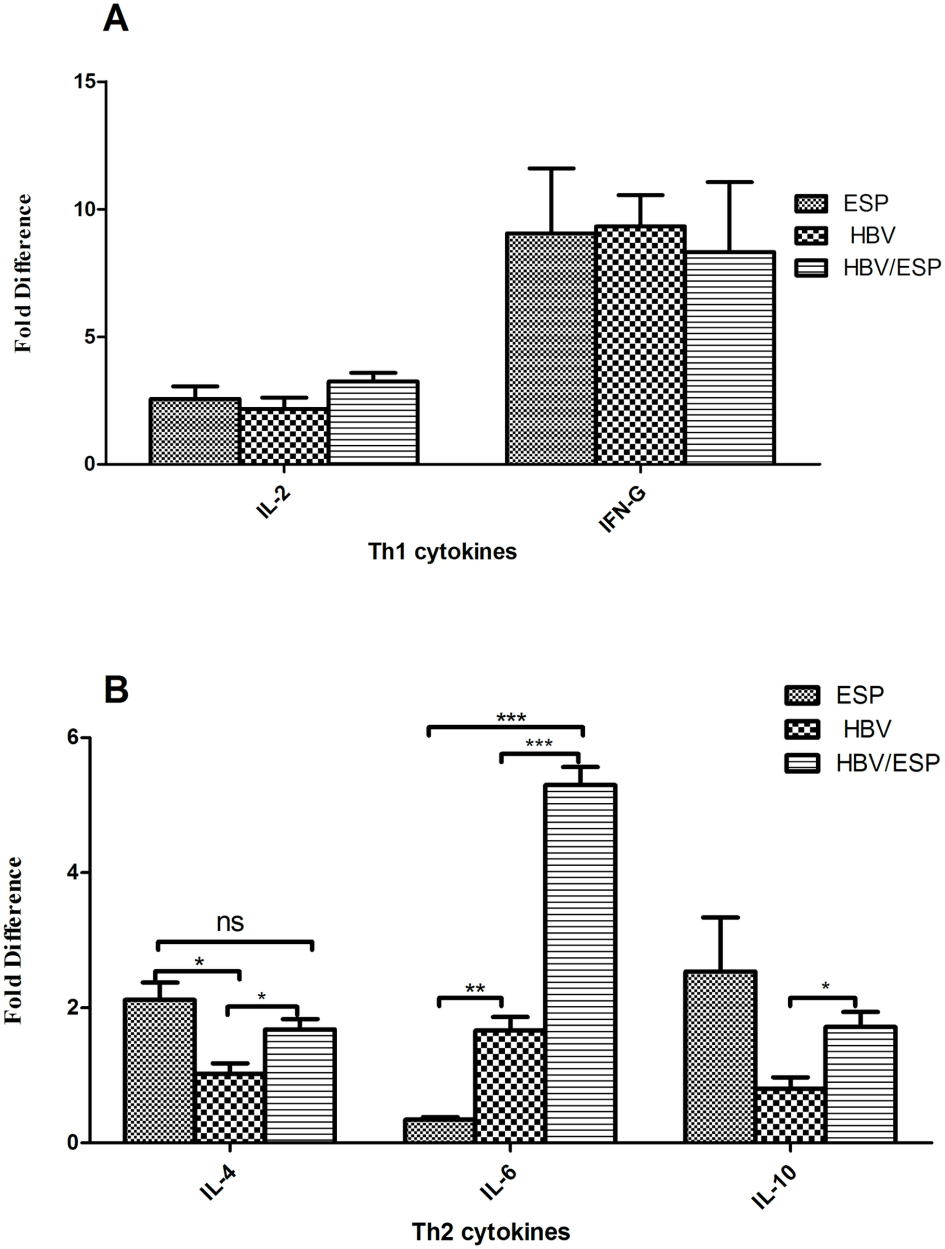
mRNA levels of Th1 and Th2 cytokines secreted by stimulated PBMCs. Total RNAs from PBMCs stimulated by mixtures of ESP and HBV positive serum, HBV positive serum only, or ESPs only were extracted for reverse transcription using cytokine gene-specific primers for Th1 cytokine (A) including IL-2 and IFN-γ and Th2 cytokines (B) including IL-4, IL-6 and IL-10 and human β-actin. The relative expression of each cytokine was detected by quantitative real-time RT-PCR and normalized relative to β-actin expression. Medium only served as a control. Data are shown as the mean ± SEM (*p <0.05, ** *p* <0.01, *** *p* <0.001).

To define and compare the secretion of Th1 cytokines (IL-2 and IFN-γ) and Th2 cytokines (IL-4, IL-6 and IL-10) following *in vitro* stimulation, we used quantitative RT-PCR to analyze the mRNA levels of each cytokine secreted by PBMCs, which were stimulated with ESPs alone, HBV positive patient serum alone or the mixture of the two, respectively. Data have been normalized for β-actin transcript expression. As showed in Fig 3, the levels of different cytokine mRNAs varied. IL-4 and IL-10 cytokine mRNA levels were higher in ESP-stimulated PBMCs than in HBV-stimulated PBMCs (Fig 3B), whereas there is no significant difference in IL-2 and IFN-γ cytokine mRNA levels between these two groups (Fig 3A). In particular, IL-6 cytokine mRNA levels were significantly higher in HBV positive sera stimulated PBMCs than in ESP-stimulated PBMCs (Fig 3B). This finding suggested that both stimulators could induce PBMCs to produce Th1 and Th2 cytokines in vitro. Furthermore, in response to the treatment with a mixture of HBV and ESPs, IL-4 and IL-10 mRNA level increased two-fold, and IL-6 mRNA level increased three-fold compared with PBMCs stimulated with HBV positive sera alone, while there were no significant changes in IL-2 and IFN-γ levels. These data suggested that PBMCs stimulated by a mixture of ESP and HBV produced higher level of Th2 cytokines including IL-4, IL-6 and IL-10 compared to HBV alone, it seems that due to presence of ESP, the cytokine production shift towards Th2.

### Cytokine profiles of the co-infected, mono-*C*. *sinensis*, and mono-HBV patients and the healthy controls

To validate the real-time PCR results, the protein levels of those cytokines were examined in cell culture supernatant both from stimulated PBMCs from healthy donors and chronic HBV patients (S1 Fig). ESP/HBV stimulated PBMCs secreted a higher level of IL-4, IL-6 and IL-10 than in the HBV group, and IFN-γ was lower in ESP/HBV than in HBV (S2 Fig). However, there was no significant difference between ESP/HBV and HBV groups with regard to IL-2 level.

A similar pattern of cytokine expression could be observed in the stimulated PBMCs from chronic HBV patients (S3 Fig). Note that there is no significant difference in IL-4 and IL-6 in stimulated PBMCs with those from chronic HBV patients. These data confirm that PBMCs stimulated by a mixture of ESPs and HBV mainly produced Th2 cytokines.

To verify whether there are changes in serum cytokine levels in *C*. *sinensis*/HBV co-infected patients, HBV mono-infected patients and *C*. *sinensis* mono-infected patients, we performed cytokine ELISA to examine the levels of IL-2, IL-4, IL-6, IL-10 and IFN-γ. *C*. *sinensis*/HBV co-infected patients had both lower IFN-γ and IL-2 levels than both HBV mono-infected and *C*. *sinensis* mono-infected patients (S2 Table). All 3 groups of infected patients had higher IL-4, IL-6 and IL-10 levels than healthy control subjects. IL-6 levels were further increased in *C*. *sinensis*/HBV co-infected patients, compared with those in HBV mono-infected patients (*p* <0.05). *C*. *sinensis*/HBV co-infected patients had higher IL-10 levels than HBV mono-infected patients (*p* <0.05).

## Discussion

In this study, we investigated the relationship between *C*. *sinensis* infection and HBV infection in humans and further studied the impact of *C*. *sinensis* infection on the efficacy of antiviral treatment.

In our study, we provide strong evidence for the existence of an association between HBV infection and *C*. *sinensis* infection. Our data showed that a correlation of co-infection *C*. *sinensis* and HBV developed predominantly in males. This finding is supported by previous reports that *C*. *sinensis* infections in male individuals are usually higher than that in female individuals [23–25]. Additionally, our results showed that co-infected patients had significantly higher liver transaminases levels as well as HBV DNA copies, indicating that concomitant *C*. *sinensis* infection aggravated the liver disease. PZQ is known to be very effective and the drug of choice against trematode and cestode infections. Mesan et al. demonstrated that oral PZQ to patients co-infected with schistosomiasis and hepatitis C virus (HCV) could help the response to HCV treatment [26]. Patients who received *C*. *sinensis* infected liver transplantation who took PZQ experienced improved liver function[27]. The present study provides ample evidence of significant decreases in the levels of TB and HBV DNA copies by using the combination of PZQ during antiviral therapies in co-infected patients. It is theorized that the beneficial effects are likely related to the clearance of *C*. *sinensis* worms and subsequent reduction of metabolites of *C*. *sinensis*. This result further indicated that the efficacy of HBV antiviral treatment was related to the removal worms in co-infected patients.

On the other hand, previous studies suggested that *Schistosoma mansoni* soluble egg antigens (SEA) have the potential to enhance HCV propagation [28] and SEA of *Schistosoma Haematobium* induces HCV replication in PBMCs [29], which indicates that some components of trematode could enhance viral replication. ESPs of *C*. *sinensis* could cause chemical damage to the host [30,31] and induce cell proliferation in vitro [32]. Other findings suggested that in vitro infection of PBMCs with human sera contain HBV particles may be a suitable model to study the early steps of the viral life cycle [33,34]. *M*. *Cabrerizo* et al showed that the viral DNA can be detected after incubation of PBMCs with human sera containing HBV particles and that HBV is able to infect, replicate and release viral particles in the medium in in vitro infected PBMCs[35]. Therefore, we have used cultures of PBMCs from a healthy donor to test the impact of ESPs on HBV particle replication. We have demonstrated that HBV DNA can be detected in the supernatant 48 h after incubation of PBMCs with human sera containing HBV particles. Additionally, the results revealed that significantly higher level of HBV DNA in the supernatant from cells co-cultured with HBV positive sera and ESPs. This may explain, at least in part, the higher HBV DNA copies observed in co-infected patients. Unfortunately, which components of ESPs are involved were not identified in this study. Additional studies are required to determine which components of ESPs are the key role players.

Studies on bile and serum of patients indicated that infection with *C*. *sinensis* correlated with Th2 type responses, emphasizing the decrease in concentration in IL-2 and an increase in IL-4. It was suggested that ESPs are immunogenic, stimulate inflammation and promote proliferation, and suppress apoptosis [32]. Most proteins belonging to ESPs, including *Cs*RNASET2, *Cs*LAP2, and *Cs*NOSIP, contributed in eliciting Th2 immune response in mice [36–38]. Cytokines are important mediators in the regulation of the immune response. However, it was not known yet whether ESPs of *C*. *sinensis* could stimulate PBMCs to produce cytokines in vitro as well as whether they have an influence on cytokine expression produced by HBV positive sera stimulated PBMCs. Thus, we examined the levels of cytokine specific mRNAs to clarify the cytokine response. We observed that PBMCs stimulated by either ESPs or HBV positive sera could prompt the secretion of Th1 cytokines by enhancing the expression of IL-2 and IFN-γ and Th2 cytokines by enhancing the expression of IL-4, IL-6 and IL-10. Additionally, in response to mixtures of HBV positive sera and ESPs, the levels of Th2 cytokines were notably higher compared to HBV positive sera alone, whereas there was no significant change in Th1 cytokine expression level, indicating that HBV/ESP predominantly produced Th2 cytokines. IL-6 exhibits both pro- and anti-inflammatory functions in innate immunity [39] and several studies have shown that IL-6 serum levels are increased in HBV positive patients, significantly higher in patients with severe and acute infections [40,41]. *Wang* et al. suggested that IL-6 is involved in the activation of natural killer cells and cytotoxic T lymphocytes induce the killing of hepatocytes, indicating that IL-6 plays an important role in liver cell necrosis and apoptosis[42]. Combined with our observation, these results suggested that a significantly higher level of IL-6 in response to a mixture of HBV and ESP stimulation, which may explain why the liver function is damaged severely in co-infected patients. In addition, compared to HBV infection alone, the levels of IL-4 and IL-10 were significantly increased, but IFN- γ was not changed in mixed HBV and ESP stimulation, indicating that cytokine production may shift from Th1 response to Th2 response. However, Th1 (including IL-2 and IFN- γ) cytokines have been identified to participate in the viral clearance while Th2 cytokine IL-10 serves as a potent inhibitor of Th1 effectors cells[43]. Therefore, one possible reason for the weaker response to antiviral therapies in co-infected *C*. *sinensis*/HBV patients was that the shift in cytokine production from Th1 to Th2 that may inhibit viral clearance. Cytokines participate in the induction and effector phases of the immune and inflammatory responses based on the protein level rather than the mRNA level; thus, we determined the protein levels of the above cytokines. Our results suggested that the cytokine pattern were similar between mRNA and protein levels. However, when we evaluated the response of PBMCs from chronic HBV patients to the same stimulators, the pattern of cytokine expression was similar to health subjects’ PBMCs, except for IL-4 and IL-6 level. We reasoned that PBMCs from chronic HBV patients may consist of antigen specific T and B cells, which may interfere with cytokine production.

IL-6 plays an important role in host defense against pathogens and mediates anti-parasite protective responses[44]. Additionally, IL-6 may participate in pathological complications of HBV [45]. Our results are consistent with these findings; we found that serum IL-6 were significantly higher in *C*. *sinensis* mono-infected patients than both HBV mono-infected and health control subjects; that IL-6 levels were higher in *C*. *sinensis*/HBV co-infected patients than in the mono-infected group as well. IL-10 is mainly involved in the regulation of inflammatory response. IL-10 can antagonize Th1 cell responses by inhibiting Th1 cell differentiation and IFN-γ production [46]. Increased levels of IL-10 are relevant to the degree of liver inflammation and lead to disease progression [47,48]. Serum IL-10 levels have been reported in patients with chronic hepatitis and cirrhosis [49] and IL-10 can restrain the host’s anti-HBV activity. In this study, the level of IFN-γ in the co-infected patient group was significantly lower than in mono-HBV patients and mono-*C*. *sinensis* patients. IFN-γ not only is a cytokine produced by Th1 and NK cells but has a critical role in the suppression of HBV replication [50,51]. Studies have shown that chronic HBV patients have a lower level of IFN-γ; so, patients may fail to develop an efficient anti-viral immune response [51,52]. In this study, the serum level of IFN-γ in the co-infected patient group was significantly lower than in the mono-HBV patients and mono-*C*. *sinensis* patients. Therefore, we assume that co-infection with *C*. *sinensis* in HBV infection may suppress the immune response by stimulating IL-10 production as well as inhibiting IFN-γ secretion as a result. In addition, our results showed that *C*. *sinensis* seems to induce Th2-related cytokines, with an increase in serum levels of IL4, IL-6 and IL-10. Given that serum cytokine levels were fluctuating in *C*. *sinensis*/HBV patients, this could partly suggest that *C*. *sinensis* infection could exacerbate the imbalances of Th1/Th2 cytokine in HBV patients; however, further analysis is warranted.

In conclusion, this study is the first to provide strong evidence for the association between *C*. *sinensis* infection and HBV infection. Co-infected individuals presented weaker liver function and higher HBV DNA titers. In co-infected patients, the efficacy of anti-viral treatment was better in patients who were prescribed ETV and PZQ than ETV alone. *C*. *sinensis*/HBV co-infection could exacerbate the imbalance of Th1/Th2 cytokine, which may lead to the chronicity of HBV infection, and *C*. *sinensis* may play a role in the unresponsiveness to antiviral therapy in co-infected patients. Further investigations are required to address this point.

## Supporting Information

S1 Table Accession numbers of genes mentioned in the text. (XLSX)

S2 Table Levels of cytokines in serum. (XLSX)

S1 Fig Eggs of *C*. *sinensis* per gram of feces determined by Kato-Katz method. Eggs of *C*. *sinensis* per gram of feces were determined by Kato-Katz thick stool smear technique in co-infected patients. Symbols show individual measurements within the patient groups, and the graphs show the means ± SD. Asterisks indicate statistically significant differences between NONPZQ and PZQ groups, as measured by paired, two-tailed Student's t-test (** *p* <0.01).
(TIF)

S2 Fig Cytokine production by stimulated healthy donors’ PBMCs in culture. Supernatant from healthy donors’ PBMCs stimulated with mixtures of ESP and HBV positive serum or HBV positive serum only, or ESPs only were harvested after 48 h and analyzed for cytokines by ELISA using commercially available kits. Cell culture only served as a control. Data are shown as the mean ± SEM (*p <0.05, ** *p* <0.01, *** *p* <0.001).
(TIF)

S3 Fig Cytokine production by stimulated chronic HBV patients’ PBMCs in culture. Supernatant from chronic HBV patients’ PBMCs stimulated by mixtures of ESPs and HBV positive serum or HBV positive serum only, or ESPs only were harvested after 48 h and analyzed for cytokines by ELISA using commercially available kits. Cell culture only served as a control. Data are shown as the mean ± SEM (*p <0.05, ** *p* <0.01, *** *p* <0.001).
(TIF)
