# Supplementary figures and images for: Clonorchis sinensis Co-infection Could Affect the Disease State and Treatment Response of HBV Patients
Source: PLoS Negl Trop Dis. 2016 Jun 27;10(6):e0004806. doi: 10.1371/journal.pntd.0004806 (PMC4922651; doi:10.1371/journal.pntd.0004806)

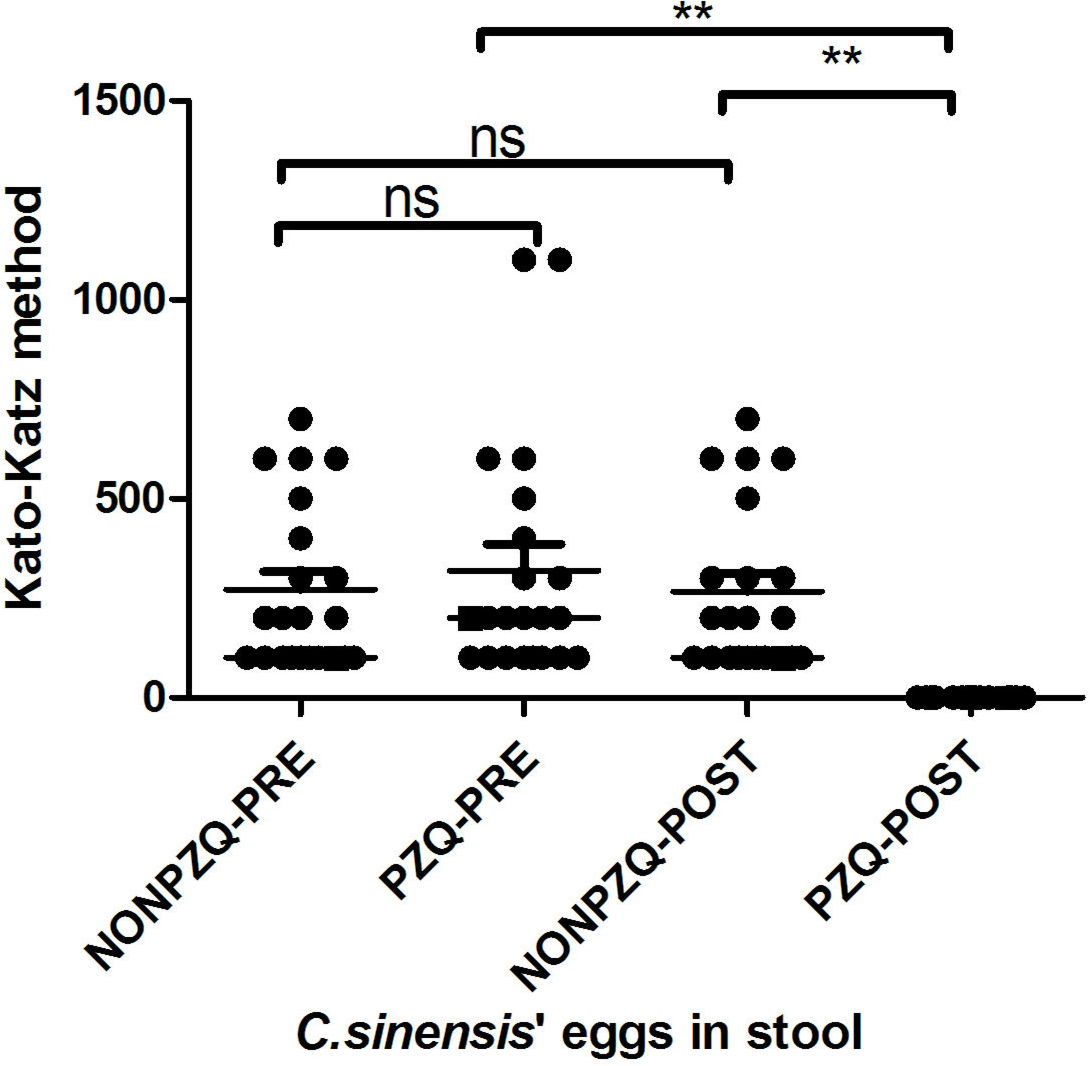

Supplement: S1 Fig — Eggs of C. sinensis per gram of feces were determined by Kato-Katz thick stool smear technique in co-infected patients. Symbols show individual measurements within the patient groups, and the graphs show the means ± SD. Asterisks indicate statistically significant differences between NONPZQ and PZQ groups, as measured by paired, two-tailed Student's t-test (** p <0.01). (TIF) [file pntd.0004806.s003.tif]

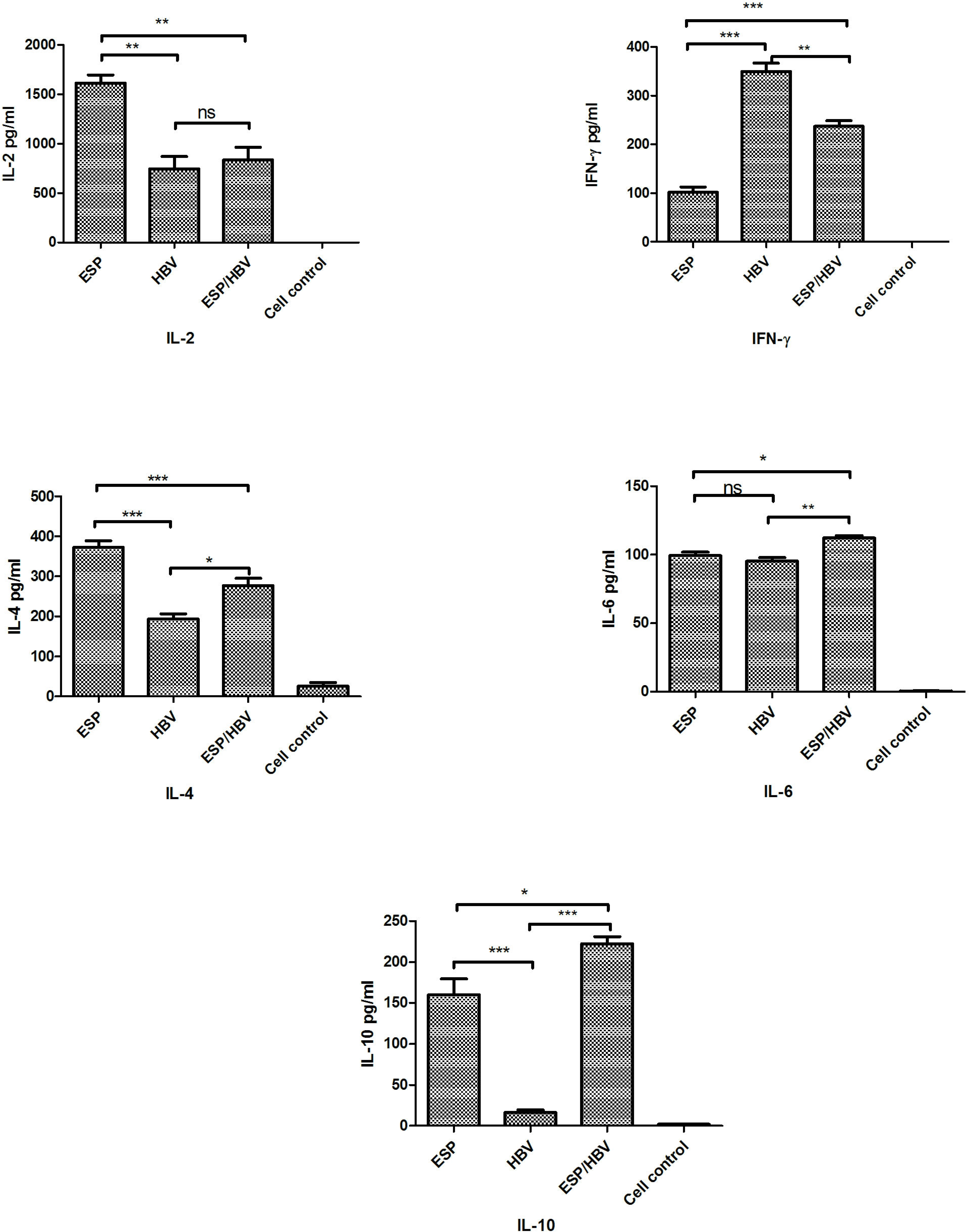

Supplement: S2 Fig — Supernatant from healthy donors’ PBMCs stimulated with mixtures of ESP and HBV positive serum or HBV positive serum only, or ESPs only were harvested after 48 h and analyzed for cytokines by ELISA using commercially available kits. Cell culture only served as a control. Data are shown as the mean ± SEM (*p <0.05, ** p <0.01, *** p <0.001). (TIF) [file pntd.0004806.s004.tif]

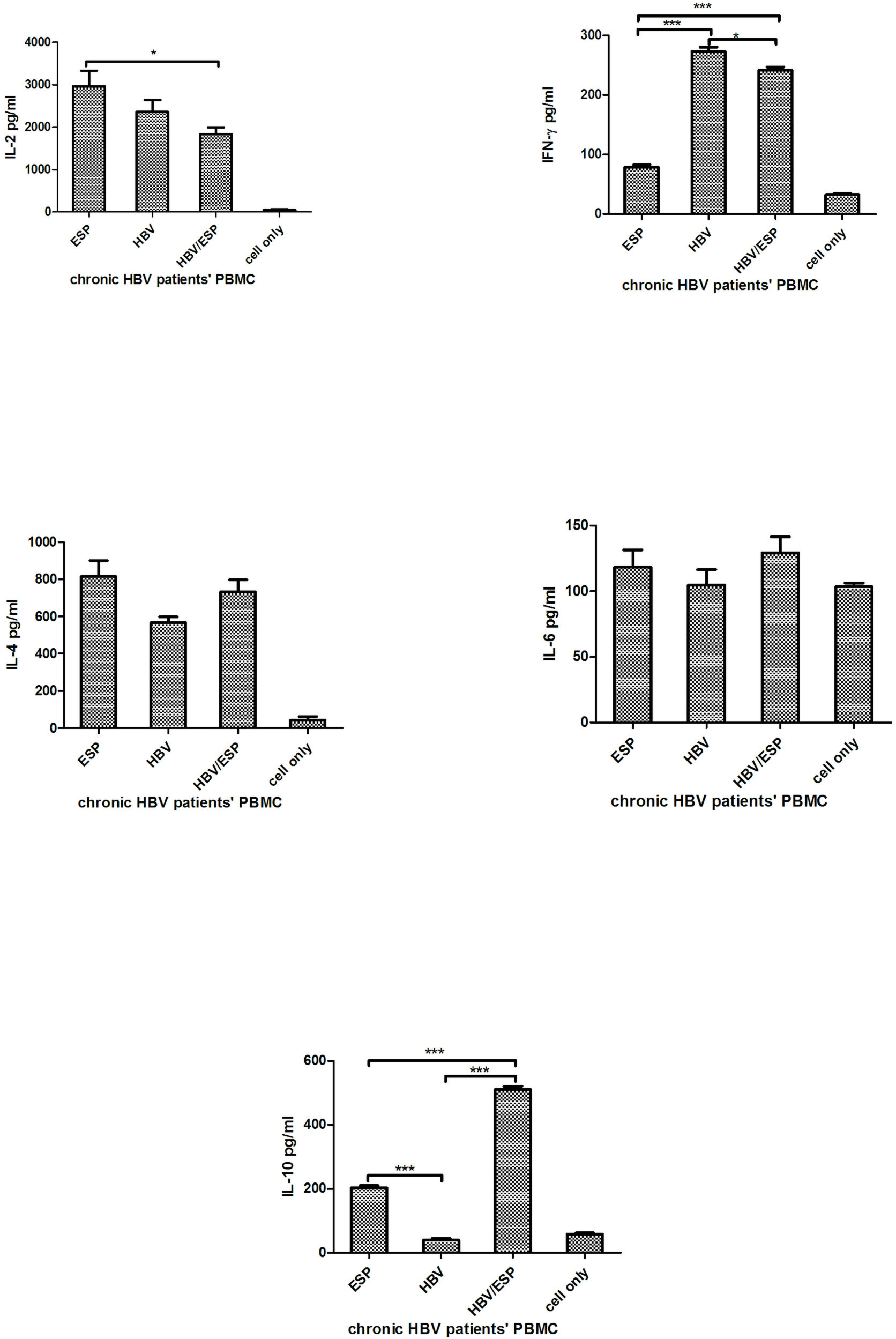

Supplement: S3 Fig — Supernatant from chronic HBV patients’ PBMCs stimulated by mixtures of ESPs and HBV positive serum or HBV positive serum only, or ESPs only were harvested after 48 h and analyzed for cytokines by ELISA using commercially available kits. Cell culture only served as a control. Data are shown as the mean ± SEM (*p <0.05, ** p <0.01, *** p <0.001). (TIF) [file pntd.0004806.s005.tif]
